# The Effect of Carbohydrate Ingestion on Performance and Indices of Fatigue in Adolescent Soccer Players During a Simulated Game

**DOI:** 10.3390/sports13060192

**Published:** 2025-06-19

**Authors:** Panagiotis G. Miliotis, Spyridoula D. Ntalapera, Dimitriοs C. Stergiopoulos, Athanasios C. Zavvos, Panagiota Klentrou, Ifigeneia Giannopoulou, Nickos D. Geladas

**Affiliations:** 1 Section of Sport Medicine and Biology of Exercise, School of Physical Education and Sport Science, National and Kapodistrian University of Athens, 17237 Athens, Greece; sntalapera@phed.uoa.gr (S.D.N.); dstergio@phed.uoa.gr (D.C.S.); atzavvos@phed.uoa.gr (A.C.Z.); i.giannopoulou@brighton.ac.uk (I.G.); ngeladas@phed.uoa.gr (N.D.G.); 2 Department of Kinesiology, Brock University, St. Catharines, ON L2S 3A1, Canada; nklentrou@brocku.ca; 3 School of Education, Sport and Health, University of Brighton, Brighton BN2 4AT, UK

**Keywords:** physical performance, carbohydrate supplementation, soccer fatigue, soccer game simulation

## Abstract

We examined the effects of carbohydrate ingestion on endurance performance and fatigue during a soccer simulation in adolescent soccer players and evaluated the protocol’s reliability. Nine (13.5 ± 0.4 years pre-PHV) soccer players performed two soccer simulation intermittent exercise sessions on the treadmill (60 min) while consuming 4 boluses of either a CHO or PLC beverage in random, counterbalanced order. Before and immediately after each exercise session, MVC was measured for the quadriceps and the hand. Participants also performed a TTE on a cycle ergometer on three occasions, after each simulation exercise session (CHO and PLC), and on another day in a rested state (CON). The simulation protocol produced an ICC of 0.96 ([0.77–0.98 95%CI], *p* = 0.01) for VO_2_, with 2.24%CV between trials, suggesting strong reliability. TTE was higher (*p* = 0.01) in the CHO condition (123 ± 33 s) compared to PLC (85 ± 5 s) by 29%. The relative reduction in MVC_LEG_ was more pronounced in the PLC (22 ± 11%) condition than in CHO (14 ± 6%) (*p* = 0.05). Compared to the PLC, CHO resulted in lower RPE_local_ during the second half of the simulation protocol (*p* < 0.05). Carbohydrate ingestion can improve endurance performance and reduce peripheral fatigue during a reliable soccer simulation that resembles the physiological demands of a youth soccer match.

## 1. Introduction

Soccer is the most favorable sport among young populations. The long duration (~60–70 min) in conjunction with high-intensity intermittent actions imposes a high physiological demand on children and adolescent soccer players [1,2,3,4]. Studies in adults using muscle biopsy have demonstrated that towards the end of a soccer game, the muscle glycogen stores are partly depleted [5,6,7], thus inducing fatigue and reducing aerobic performance [8,9,10,11]. Carbohydrate ingestion spares glycogen utilization, thereby enhancing exercise performance in adults [12,13]. Despite its potential benefits, limited research has investigated the effects of carbohydrate ingestion during a soccer-specific running activity in adolescents. One such study in adolescents found that carbohydrate ingestion improved endurance capacity by 24% during prolonged high-intensity intermittent exercise compared to a placebo condition [14]. However, this study included participants of both sexes and of a broad range of chronological age (12–14 years) and maturity (age from Peak Height Velocity [PHV] −1.51 to +1.27 yrs). It is suggested that biological maturation and sex may be confounding factors when examining the ergogenic effect of carbohydrate supplementation in adolescents [15,16]. Indeed, pre-pubertal boys have been found to oxidize more exogenous carbohydrates than mid- to late-pubertal boys, independent of chronological age [17]. This variation in substrate utilization, due to maturation and chronological age, highlights the importance of controlling for maturation and sex when investigating the effects of supplementation in youth athletes and the need for further research on carbohydrate supplementation, exercise performance, and fatigue in selected maturation stages.

In the context of fatigue, adult studies have shown that carbohydrate supplementation during exercise is linked to the preservation of mental state, reduced sensation of effort, and contained decline of neuromuscular performance [18,19,20]. However, the effect of carbohydrates specifically on neuromuscular performance in pre-PHV youth soccer players is less documented, although children and adolescents manifest greater fatigue resistance during short-duration whole-body exercise (i.e., repeated sprint) compared to adults, which is also inversely related to biological maturation [21,22,23]. Also, in youth soccer players, recent data suggests that the magnitude of the acute neuromuscular response may be associated with maturation during competitive soccer games [24]. Despite that knowledge, there is a scarcity of studies investigating the effect of carbohydrate ingestion on indices of fatigue during prolonged exercise in youth athletes, and findings regarding perception of effort remain equivocal [14,25,26].

The aim of the present study was to examine the effects of carbohydrate ingestion during a 60 min treadmill-based simulated soccer game protocol on measures of performance and indices of fatigue in pre-PHV adolescent soccer players. In most studies, the participants exercised under absolute speed thresholds that may induce disparity in physiological load among participants. As a result, we designed and tested the reliability of a new laboratory-based soccer simulation exercise protocol on a treadmill, which aimed to produce the same metabolic demands across participants and to closely mimic the internal and external load of an official youth soccer game [1]. We hypothesized that, compared to a placebo condition, carbohydrate supplementation would improve the time to exhaustion during a performance test and would reduce neuromuscular fatigue.

## 2. Materials and Methods

### 2.1. Participants

Nine healthy adolescent male soccer players volunteered to participate in the study. An a priori power analysis (G Power 3.1 software) indicated that a minimum of six participants was required to achieve statistical power greater than 0.80, effect size > 0.80, and a value (≤0.05) for repeated measures analysis. All participants were pre-PHV (Table 1). Maturation status was determined by the somatic maturity offset estimated using a non-invasive equation that includes somatometric measurements of participants [maturity offset = −8.128741 + (0.0070346 × (age × sitting height))] [27]. This gender-specific equation estimates the years from PHV within an error of ±1 y 90% of the time. Height and sitting height were measured to the nearest mm using a stadiometer (Bilance Salus, Milan, Italy), and body mass was measured to the nearest 0.1 kg on a standard scale (Bilance Salus, Milan, Italy). A negative value indicates years from reaching PHV, and a positive value indicates years since the participant attained PHV. Participants were recruited from a soccer club. The inclusion criteria were being 13 and 14 years old, pre-PHV, highly trained with more than 3 years of experience, a minimum of 4 training sessions per week plus an official soccer game, and free from any musculoskeletal injury within the past 2 months. The study was approved by the Research Ethics and Bioethics Committee of the School of Physical Education and Sport Science, Athens, Greece (1524/17-05-2023).

### 2.2. Reliability of Soccer Simulation Exercise Protocol

**Figure 1:**
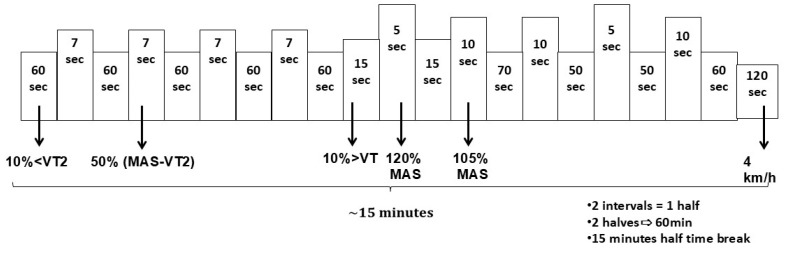
Protocol simulation of youth soccer match play using individualized speeds. A total of 10% < VT2: below of VT2, 10% > VT2: above of VT2, 50% (MAS-VT2): 50% of the difference between the velocity of VT2 and MAS (Maximal Aerobic Speed). 120% & 105% MAS: supramaximal speed above MAS.

The reliability of the soccer simulation protocol was examined in an additional group (Table 1) of seven healthy young participants who visited the lab on two separate occasions (DAY 1 and DAY 2) to perform two prolonged (~60 min) high-intensity interval exercise trials (Figure 1). Trials were scheduled at least 5 days apart, and the participants were instructed to avoid intense exercise 48 h before each trial. Both trials were completed within a month of the first visit.

### 2.3. Study Design

This was a cross-over, randomized, double-blind controlled study, where each participant performed two experimental, intermittent exercise sessions simulating a soccer match while consuming either a 6% carbohydrate (CHO) or a flavored placebo (PLC) beverage in random and counterbalanced order. Specifically, participants reported to the lab on 4 occasions. During visit 1, they performed an aerobic peak test (V̇O_2peak_), a Wingate anaerobic test (WAnT), and were familiarized with the intermittent exercise protocol that simulates a soccer match. A day later, participants came for their second visit to perform a time-to-exhaustion test without treatment, which served as the control session (CON). Following 5 days of rest, the volunteers returned to the lab for the two experimental sessions, which involved a treadmill-based soccer simulation exercise consuming 4 boluses of either the CHO or the PLC beverage. The experimental sessions were scheduled at least 5 days apart, and the volunteers were instructed to avoid intense exercise 48 h before sessions.

### 2.4. Treatment Administration

The experimental sessions were performed from 10.00 am to 12.00 pm when official matches are usually played. Participants were asked to record their diet 24 h before the first session and repeat the same diet the day before the next experimental session and were analyzed using Nutritics Professional Edition software v4.315 (Dublin, Ireland). They were also required to consume a standardized breakfast 2.5 h before each experimental session. The standardized breakfast (milk, cereal, banana) consisted of 2.2 g/kg CHO. During the experimental sessions, participants performed the soccer simulation exercise protocol on the treadmill, consuming either a 6% (glucose) CHO or a flavored PLC beverage in a randomized and counterbalanced order. Specifically, they consumed 4 boluses of the appropriate beverage: the first bolus (5 mL/kg) 10 min before exercise and 3 boluses (2 mL/kg) at 15 min intervals during exercise.

### 2.5. Baseline Exercise Testing

The participants performed an incremental exercise test to exhaustion on a treadmill (Technogym, Cesena, Italy) to determine their aerobic capacity. Gas exchange and pulmonary ventilation were recorded continuously, breath by breath, via open-circuit spirometry (MedGraphics, CPD-X, Austin, TX, USA). The initial speed was set at 5 km/h, then increased by 1 km/h every minute until volitional fatigue. Verbal encouragement was given by researchers throughout testing. V̇O_2peak_ was considered as the mean value during the last 20 s of the exercise when at least one of the following criteria was attained: (1) heart rate ± 10 b/min of maximum (HRmax) defined as 220-age; (2) respiratory exchange ratio (RER) > 1.15; (3) failure to maintain running speed in the last stage; (4) RPE at termination > 8 [28,29]. The maximal aerobic speed (MAS) that was achieved during the test was recorded to be used in the experimental sessions. The second ventilatory threshold (VT_2_) was determined according to end tidal values (P_ET_O_2_, P_ET_CO_2_), pulmonary ventilation equivalents (V̇_E_/V̇O_2_ and V̇_E_/V̇CO_2_), and V̇_E_ [30,31]. Furthermore, as part of a preliminary examination, the volunteers performed a Wingate Anaerobic Test (WAnT) on a cycle ergometer (Monark 650E, Vansbro, Sweden). The WAnT test lasted 10 s to determine the maximum amount of power (Pmax), which is normally attained during the first 5–6 s of the test [32]. The resistance was set at 7.5% of individual body mass and applied after an unloaded acceleration phase lasting 3 s at 70 rpm. The WAnT was performed approximately 20 min or longer before the V̇O_2peak_ test.

### 2.6. Experimental Procedure

During both experimental sessions, i.e., CHO and PLC, participants performed the same 60 min soccer simulation exercise protocol that was designed to produce the same physiological load among participants. This was achieved using individualized intensities based on MAS and VT_2_ achieved in the baseline exercise testing (Figure 1). The corresponding intensities for each participant were defined using the following classification criteria: at 105% of MAS and at 120% of MAS as supramaximal intensities, at 50% of speed difference between MAS and the velocity at VT_2_ (vVT_2_) as very high intensity, 10% below and above the VT_2_ as moderate and high intensities, respectively, and walking speed of 4 km/h as low intensity. Time duration and number of repetitions at each intensity were adjusted so that the athletes were able to withstand high-intensity exercise for prolonged periods, like a real soccer game. The simulation exercise protocol was split into two halves, each consisting of 2 intervals each lasting 12:45 min. However, due to the time delay to achieve the corresponding speed, the overall duration of each interval was approximately 15 min (Figure 1). Between exercise intervals, there was a 15 min break.

### 2.7. Fatigue Measurements

During both experimental sessions, the rate of perceived exertion (RPE) was recorded as a dyspnea discomfort (RPE_Central_) and as a leg discomfort (RPE_Local_) sensation of fatigue [33] with the OMNI scale (0–10) of perceived exertion [34]. Before and immediately after (<3 min) the simulation exercise session, a maximal voluntary contraction (MVC) test of the quadriceps (MVC_Leg_) was performed as an index of peripheral fatigue. The MVC_Leg_ test was performed for knee extensors on a modified strength training device, at a knee angle of 60° (0° = fully extended) [35]. Participants were stabilized using elastic belts. The participant’s leg was stabilized using a custom-designed chain system connected to a specialized ankle brace. This setup restricted the lower limb from extending below 60 degrees of knee flexion. In addition, the MVC of handgrip (MVC_HAND_), a non-exercised muscle group, was used as an index of central fatigue [36]. The MVC_HAND_ was executed in the dominant hand with the elbow flexed at 90°, in a sitting position. For both measurements, the participants executed 3 maximal voluntary contraction trials lasting 3–5 s with 1 min rest between trials. The mean value of the two highest trials was taken as the maximum voluntary contraction. The data were recorded by a force cell (LC-500F, Kyowa, Japan) and by an isometric dynamometer (SS-25, BIOPAC Systems Inc., Goleta, CA, USA). The signal was transmitted from the mobile data collection unit (ΤΕL 100D, BIOPAC Systems, Inc., Goleta, CA, USA) to the signal processing unit (ΜΡ 100A, BIOPAC Systems, Inc., Goleta, CA, USA). The signal was recorded in volts and subsequently converted to Newtons.

Following the post-simulation protocol measurement of MVC_Leg_ and MVC_Hand_, a time to exhaustion (TTE) test was performed on a cycle ergometer (Lode, Groningen, Netherlands) rather than on the treadmill to avoid any accidental fall due to fatigue or discomfort. The intensity of this test corresponded to ~40% of Pmax from the WAnT, which has been previously examined in a pilot group of 7 participants and showed an excellent mean within-subject coefficient of variation (CV%) of 4.9%. The pedal rate was set at 70 rpm, and the participants started pedaling initially without a load. Thereafter, within 5 s, the required workload was applied, and immediately the time until exhaustion began to be recorded. Throughout the test, two researchers verbally encouraged the participants until exhaustion, which was defined as the inability to maintain the pedal rate above 50 rpm. In addition to the two experimental sessions, during which participants ingested either CHO or PLC and subsequently performed the TTE test following the simulation protocol, they also attended a third session. In this session, they performed only the TTE test without preceding it with the simulation protocol (CON).

### 2.8. Statistical Analysis

#### 2.8.1. Reliability of Soccer Simulation Exercise Protocol

Paired *t*-tests were used to examine differences in V̇O_2_, RER, HR, and total distance covered during simulation between DAY 1 and DAY 2. In order to quantify agreement between the two measurements of V̇O_2_ during the simulation trials, we performed a Bland–Altman analysis [37,38] plotting the difference of the two paired measurements against the average of the two measurements, with the recommendation that 95% of the data points would lie within ±1.96 s of the mean difference. Also, test-retest reliability was examined using Intraclass Correlation Coefficient analysis (ICC, model 3,1), with 95% confidence intervals (95% CI). Also, the within-subject coefficient of variation (%CV) was assessed between 2 trials: $CV\left(\%\right)=\frac{TE}{Mean}\times 100$ where *TE* is a typical error. The significance level was set at ≤0.05.

#### 2.8.2. Carbohydrate Ingestion

The normality of all variables was confirmed using the Shapiro–Wilk test. One-way repeated measures ANOVA was conducted for the TTE performance test. Two-way ANOVA for repeated measures (session/condition × time) was performed to test the significance between and within conditions (CHO and PLC). Following a significant F test, pairwise differences were identified using Tukey’s post hoc analysis. In addition, a series of paired *t* tests were used to examine differences in total distance covered, ΔMVC, performance index, HRmax, and RPE at the point of exhaustion during TTE between CHO and PLC conditions. Furthermore, Cohen’s effect size was calculated for exercise performance and fatigue-related comparisons, where d = 0.2 is considered small, 0.5 is moderate, and >0.8 is large [39]. The level of significance was set at 0.05. The analysis was conducted on IBM software SPSS v25.

## 3. Results

### 3.1. Reliability of Soccer Simulation Exercise Protocol

**Figure 2:**
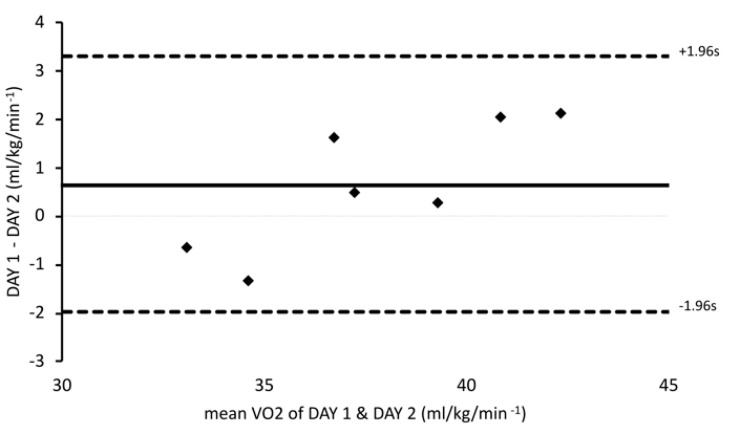
Differences between DAY 1 and DAY 2 plotted against mean V̇O_2_ of the two measurements, with limits of agreement from −1.96 s to +1.96 s (dotted line).

Average V̇O_2_ (mL/kg/min) was not significantly different between simulation trials (DAY 1: 38.06 ± 3.9 vs. DAY 2: 37.40 ± 2.8; *p* = 0.24). Likewise, HR (bpm) response was similar between DAY 1 and DAY 2 (167.8 ± 5.7 vs. 166.5 ± 3.7, respectively; *p* = 0.48), corresponding to ~84% of HRmax. RER also did not differ between trials (DAY 1: 0.96 ± 0.3 vs. DAY 2: 0.96 ± 0.3; *p* = 0.8). The average intensity during the simulation trials corresponded to 71% and 70% of V̇O_2peak_ for DAY 1 and DAY 2, respectively. The total distance covered did not differ between DAY 1 and DAY 2 (8073 ± 547 m vs. 8048 ± 523 m, respectively; *p* = 0.43). The ICC (95% CI) was 0.96 (0.77–0.99; *p* = 0.01), indicating a very strong correlation between trials, while the %CV for mean V̇O_2_ (mL/kg/min) between trials was 2.24%, suggesting very low variability. Finally, to assess between-measurements differences, the Bland–Altman analysis (Figure 2) supports the above findings since 95% of points are within the ±1.96 s of the mean difference with a very low (0.65 mL/kg/min) systematic bias.

### 3.2. Carbohydrate Ingestion

There were no significant differences between the two experimental sessions in total distance covered (CHO: 8524 ± 824 m, PLC: 8627 ± 717 m; *p* = 0.416), indicating a similar external load. In terms of internal load. Likewise, the mean heart rate response (%HRmax) was not different between conditions (CHO: 86.3% vs. PLC: 86.9, *p* > 0.05), and within conditions, the mean heart rate was not different between halves (*p* > 0.05). Moreover, the recorded diet the day before the experimental sessions was not statistically different between CHO and PLC (*p* = 0.214), PLC: 1466 kcal (carbohydrates: 49.6%, protein: 20.1%, fat: 30%), CHO: 1428 kcal (carbohydrates: 50.8%, protein: 21.2%, fat: 27.7%).

### 3.3. Exercise Performance and Fatigue Indices

**Figure 3:**
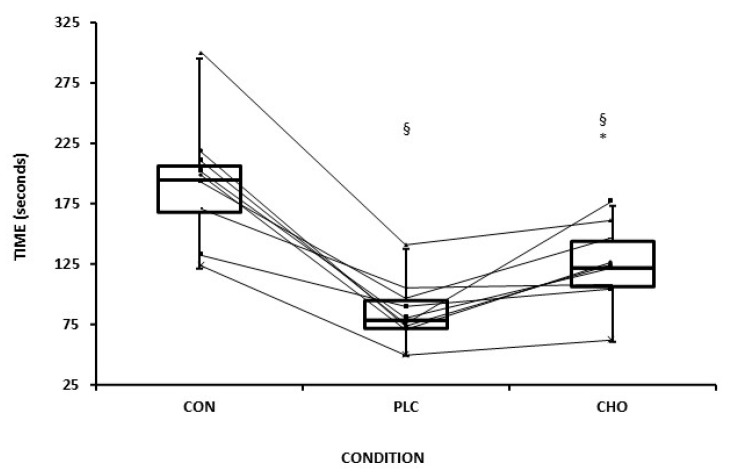
Individual responses (n = 9) to TTE (seconds) on cycle ergometer. CHO: carbohydrate fluids (n = 8), PLC: placebo fluids (n = 8), and CON: control (n = 9). §: significantly lower than CON (*p* < 0.001). * Significantly higher for CHO than PLC (*p* = 0.01). Data are presented as mean ± standard deviation.

**Figure 4:**
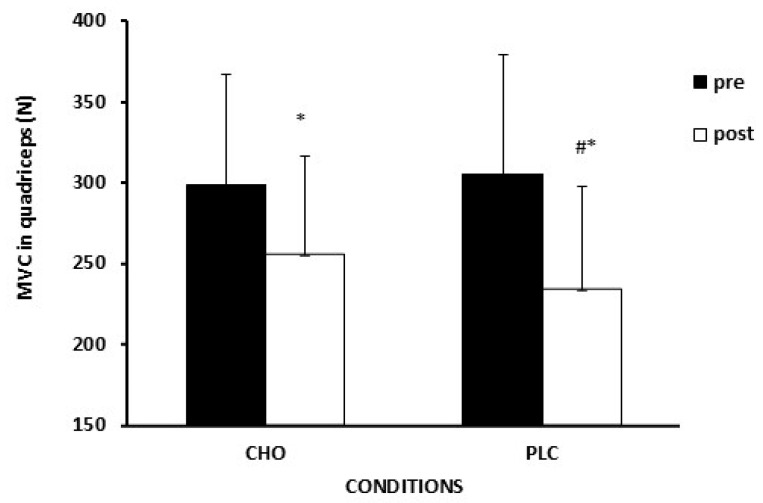
Force values in quadriceps during MVC (n = 8). * *p* < 0.05 significant reduction for post-exercise values compared to pre-exercise. # *p* = 0.05. The percentage of reduction was higher for PLC (22 ± 11%) than for CHO (14 ± 6%) condition. There was missing data for 1 subject due to technical reasons. Data are presented as mean ± standard deviation.

TTE (Figure 3) was significantly higher (*p* = 0.01, *d* = 1.22) in the CHO (123 ± 33 s) compared to PLC (85 ± 5 s) by 29 ± 18%. In addition, in the CON session, TTE (191 ± 51 s) was higher compared to both CHO (*p* < 0.01, *d* = 2.14) and PLC conditions (*p* < 0.01, *d* = 2.81). The initial force values for maximal isometric strength for quadriceps (Figure 4) before the simulation protocol were similar between CHO and PLC whereas were reduced significantly (CHO: pre:299 ± 68 Ν vs. post: 256 ± 61 Ν; PLC: pre: 314 ± 74 Νvs post: 243 ± 65 Ν) in both experimental conditions in post measurements (*p* < 0.001). However, the percentage of reduction (Δ MVC_LEG_) was more pronounced in the PLC (22 ± 11%) condition than in CHO (14 ± 6%) (*p* = 0.05, *d* = 0.80). Regarding MVC_HAND,_ there was a trend for a significant reduction (*p* = 0.06) for both CHO and PLC with no significant difference between conditions (CHO: 10% vs. PLC: 9%, *p* > 0.05).

### 3.4. Rating of Perceived Exertion

**Figure 5:**
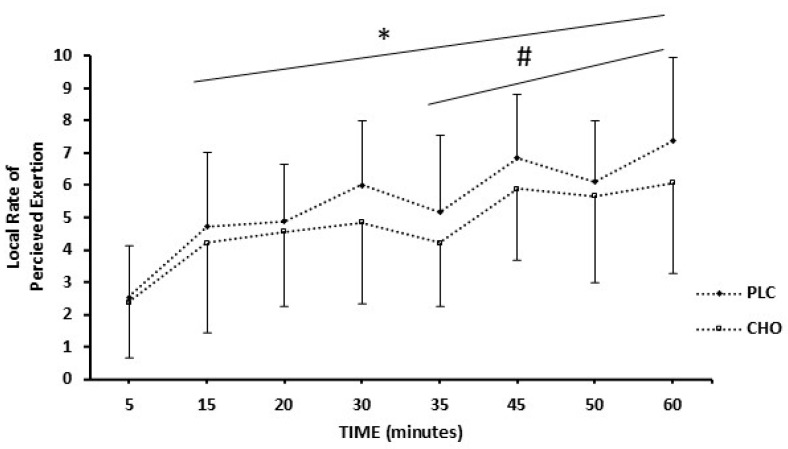
RPE_local_ during soccer simulation protocol for CHO and PLC conditions (n = 9). *: significantly higher than 5th minutes of exercise (*p* < 0.05) for both conditions. #: Significantly lower during the second half for CHO condition compared to PLC (*p* < 0.05), (n = 9). Data are presented as mean ± standard deviation.

There was a progressive increase for RPE_central_ during the exercise simulation protocol in both conditions, with average values during the simulation protocol 4.6 ± 2.0 for CHO and 5.0 ± 2.0 for PLC (*p* < 0.05). CHO ingestion led to a lower RPE_local_ (Figure 5) compared to PLC during the second half of the simulation protocol (*p* = 0.042, *d* = 0.94). At the point of exhaustion during the TTE test, RPE_local_ (PLC: 9 ± 1.2; CHO: 9 ± 1.3), RPE_central_ (PLC: 7 ± 2; CHO: 7 ± 1.6), and HRmax (PLC: 185 ± 14; CHO: 186 ± 13) were not significantly different between conditions (*p* > 0.05) despite a remarkably longer exercise time during the CHO condition.

## 4. Discussion

This study examined the role of carbohydrate ingestion on measures of performance and fatigue, in adolescent pre-PHV male soccer players during prolonged high-intensity intermittent exercise on a treadmill that simulates the metabolic demand of a youth soccer match. The main findings were: (1) CHO ingestion increased TTE during a performance test on a cycle ergometer by 29%; (2) peripheral fatigue, as determined by a reduction in MVC_LEG,_ was contained post-CHO compared to post-PLC (CHO: ~14% and PLC: ~22% reduction, respectively); (3) peripheral sensation of perceived fatigue (RPE_LEG_) was lower with CHO than PLC at specific time points during soccer match simulation; (4) the simulation protocol produced high metabolic and external load and was proved to be reliable.

When exercise intensity is at 70–80% of V̇O_2max_ and the duration of exercise is longer than 60 min, the contribution of carbohydrates as fuel is increased [12]. Accordingly, in adults, at the end of a soccer game, glycogen stores in muscle fibers are either partly or completely depleted [6,40]. Thus, carbohydrate supplementation is needed for the sparing of skeletal muscle glycogen, since glycogen depletion and hypoglycemia are infeasible for sports with a duration of 60–90 min [41]. In our study, the exercise intensity corresponded to ~71% of V̇O_2peak_ and 84% of HRmax, effectively replicating the metabolic demands of an official soccer game [1,42]. Additionally, the total distance covered was at the upper level of the distance covered during actual play, according to the literature for midfielders and wide midfielders [43,44,45]. Furthermore, the high RER values (0.96 ± 0.3) indicated that the athletes relied more on carbohydrate oxidation, reinforcing that this protocol elicited great metabolic demands and stressed the glycogen stores of athletes. Accordingly, a significant 29% improvement in TTE was observed with CHO ingestion compared to the PLC condition, possibly due to glycogen sparing.

Age, maturity, and sex play a significant role in substrate utilization during exercise. One of the strengths of our study was that we controlled these confounding factors. We included only male adolescents of the same age and before the onset of puberty in terms of somatic maturity (age from PHV = −1.4 ± 0.3 y). The first study that examined the effect of carbohydrate supplementation on performance during intermittent exercise in adolescents was conducted by Phillips and colleagues (2010). However, the results of their study may have been confounded by a wider range of maturity offset (age from PHV ranging from −1.51 to + 1.27 y) and the inclusion of both sexes (10 males, 5 females) [14]. Indeed, there are sex differences in substrate utilization in children compared to adults during exercise [17,46]. In addition, these differences are related to biological maturation, considering that pre- and early pubertal boys oxidize more exogenous carbohydrates independent of chronological age [17]. Hence, the control of biological maturation and sex in the examination of physiological responses and exercise performance is required. After controlling for sex and maturation, our results (29% improvement with CHO) align with existing literature on carbohydrate supplementation during exercise, showing improvements of approximately 30–50% in adults and 24% in adolescents [14]. Therefore, it is plausible that exogenous carbohydrates may contribute to enhanced muscle glycogen sparing or potentially support glycogen resynthesis, which is critical during the later stages of the game when glycogen stores are partly depleted [6,14]. Moreover, while previous studies reported no significant differences [14,26,47], or even higher RPE [25] values, with carbohydrate supplementation, this is the first study to demonstrate lower local perception of effort (RPE_local_) in the CHO condition compared to PLC. However, no differences were detected regarding RPE_central_. This discrepancy may be attributed to a potentially underexplored distinction in perceptual responses.

In both CHO and PLC conditions, MVC_LEG_ was reduced significantly, while in the PLC condition, this reduction was more pronounced than in the CHO condition (22% versus 14%, respectively). According to previous studies, the average decrease in MVC after a soccer match in adults ranges from 9 to 11% [48,49,50]. Our study showed greater MVC_LEG_ reduction, and this could be partly attributed to different methodologies assessing MVC (i.e., different knee angle, isokinetic dynamometer), keeping in mind that the mechanisms of fatigue are less studied in pre-PHV male players during prolonged exercise. According to the literature, children are more fatigue-resistant than adults during short-term (a few minutes), high-intensity exercise [21,23,51]. However, there is a lack of studies on the role of maturation regarding the level and the origin of fatigue during a soccer game in young players. Taken together, in our study, the simulation protocol resulted in greater fatigue in pre-PHV soccer players than the typical fatigue reported in adults following a soccer game.

The origin of fatigue may be central, peripheral, or both [52]. In adults, there is an indication that the reduction in MVC of the quadriceps after a soccer match has a central origin when examined using nerve stimulation [50,53,54]. In the present study, it is possible that the origin of fatigue was peripheral. This can be explained indirectly by examining the local and central RPE, and by the MVC_Hand_ as a non-exercised limb, and thus, as an indicator of central fatigue [36]. Our participants experienced a greater rate of peripheral (RPE_local_) than central (RPE_central_) fatigue during the soccer simulation protocol, while MVC_Hand_ was not different between CHO and PLC conditions. Also, RPE_local_ was reduced with CHO ingestion in our adolescent players compared to the PLC condition. There was a trend (*p* = 0.06) for a significant reduction in MVC_HAND_ in both groups, but this test does not directly measure central fatigue. Thus, more research is needed using more direct indices of peripheral versus central fatigue in adolescents during team sports participation.

The soccer simulation protocol, which was developed for the purposes of the present study on a treadmill, has shown high reliability in young athletes according to %CV and ICC indexes and Bland–Altman analysis. The 0.96 ICC with a good to excellent range of reliability according to the 95% CI (0.77–0.99) and very low variability (2.24%CV), proved that the simulation protocol is reliable with a very low systematic bias based on the Bland–Altman analysis. As such, this protocol could be widely used by sports scientists and coaching staff for several research and practical purposes in young athletic populations, especially those in team sports. The high metabolic load that was produced may be useful for investigating performance, and nutritional interventions, as well as for studying recovery methods and physiological responses that could be affected by training, environment, and maturation.

Since this study represents the first to examine the effects of CHO ingestion on performance and indices of fatigue using a simulation protocol in youth soccer players, the results need to be interpreted with caution, considering its limitations. One important limitation is the small sample size, making it difficult to generalize the results. Also, the simulation protocol replicates the running activity (i.e., high-speed, total distance covered), but not the specific movements of a match (i.e., change of direction, backward steps). Furthermore, the fatigue measurements, especially those aimed at investigating central origin, were indirectly assessed. Given that fatigue is a multifactorial process, future studies would benefit from employing more direct methods to evaluate fatigue components.

## 5. Conclusions

In conclusion, after controlling for sex and somatic maturity, carbohydrate ingestion improved TTE by 29%, restrained the reduction in peripheral fatigue (MVC_LEG_), and improved the sensation of local fatigue during a soccer simulation exercise protocol in a group of pre-PHV soccer players. There is a need for studies regarding the origin of fatigue during a soccer game across maturation in both sexes. The simulation exercise protocol was reliable, and its intensity and total distance covered were comparable to an official game of this age group.

## Figures and Tables

**Table 1:** Anthropometric and physiological characteristics of participants for carbohydrate ingestion (*n* = 9) and simulation protocol (*n* = 7). Values are mean ± standard deviation (range).

	Carbohydrate Ingestion (n = 9)	Simulation Protocol (n = 7)
Age (yrs)	13.4 ± 0.4 (12.8–14)	13.7 ± 0.2 (13.3–13.9)
Height (cm)	159.6 ± 6 (146–168)	153 ± 4 (148–156)
Weight (kg)	51.8 ± 4 (44.1–55.9)	45 ± 6 (42.1–53.8)
V̇O_2peak_ (L/min)	2.9 ± 0.4 (2.36–3.43)	
V̇O_2peak_ (mL/kg/min)	55 ± 5.7 (49.3–65.8)	53.5 ± 4 (50.2–59.7)
vVT_2_ (km/h)	11.3 ± 1.5 (10–14)	12.2 ± 2 (9.5–15)
MAS (km/h)	14.3 ± 0.9 (14–16)	15.9 ± 0.5 (14.1–17.5)
Pmax (w/kg)	9.8 ± 0.4 (9.35–10.52)	
Body fat (%)	10.4 ± 2 (8.8–13.1)	10.6 ± 4 (7.3–13.9)
PHV (yrs)	−1.4 ± 0.3 (−1.09 to −2.01)	−1.01 ± 0.3 (−0.8 to −1.5)

V̇O_2_peak: Peak oxygen consumption, VT_2_: Second Ventilatory Threshold, vVT_2_: Velocity at VT_2_, MAS: Maximal aerobic speed, Pmax: maximum power during Wingate anaerobic test, PHV: Peak height velocity.

## Data Availability

The data supporting the findings of this study are available from the corresponding author upon reasonable request.

## Abbreviations

The following abbreviations are used in this manuscript:

CHO	Carbohydrate ingestion condition
PLC	Placebo condition (no carbohydrates)
MVC	Maximal Voluntary Contraction
TTE	Time To Exhaustion endurance performance test
PHV	Peak Height Velocity
CV	Coefficient of Variation
ICC	Intraclass Correlation Coefficient
